# Case report: Laparoscopic gastrojejunostomy for duodenal atresia with situs inversus and preduodenal portal vein: a report of two cases

**DOI:** 10.3389/fped.2023.1220393

**Published:** 2023-06-27

**Authors:** Yoichi Nakagawa, Wataru Sumida, Satoshi Makita, Hiroo Uchida, Akinari Hinoki, Chiyoe Shirota, Takahisa Tainaka, Kazuki Yokota, Hizuru Amano, Akihiro Yasui, Aitaro Takimoto, Daiki Kato, Takuya Maeda, Yousuke Gohda

**Affiliations:** ^1^ Department of Pediatric Surgery, Nagoya University Graduate School of Medicine, Nagoya, Japan; ^2^ Department of Rare/Intractable Cancer Analysis Research, Nagoya University Graduate School of Medicine, Nagoya, Japan

**Keywords:** duodenal atresia, preduodenal portal vein, situs inversus, gastrojejunostomy, laparoscopy

## Abstract

Congenital duodenal atresia with situs inversus is occasionally accompanied by a preduodenal portal vein (PDPV), which is incidentally diagnosed during surgery. Duodenoduodenostomy is the most common and effective treatment. However, some patients require other anastomoses. Here, we present two cases of laparoscopic gastrojejunostomy for congenital duodenal atresia with situs inversus and PDPV and describe the reason for selecting gastrojejunostomy. The optimal surgical strategy is patient specific and should be determined based on the patient's general and physical condition.

## 1. Introduction

Duodenal obstruction causes bilious vomiting and requires early diagnosis and treatment. It consists of duodenal atresia and stenosis, which occur in 1 in 5,000–10,000 live births (1). Duodenoduodenostomy or duodenojejunostomy is performed in such cases to connect the intestines. Since Bax et al. originally reported performing a laparoscopic duodenoduodenostomy in 2001 (2), laparoscopic surgery for duodenal atresia has been further developed. A recent systematic review revealed that laparoscopic surgery for duodenal atresia is safe and yields outcomes equivalent to those of open surgery (3).

An appropriate strategy for the safe performance of duodenal atresia surgery is important, as more than 50% of patients with duodenal atresia have associated congenital anomalies (4). We encountered two cases of duodenal atresia with situs inversus and a preduodenal portal vein (PDPV). Reports of laparoscopic management of such cases are rare. Therefore, we present our surgical strategy for duodenal atresia to prevent postoperative complications.

## 2. Case presentation

**Figure 1:**
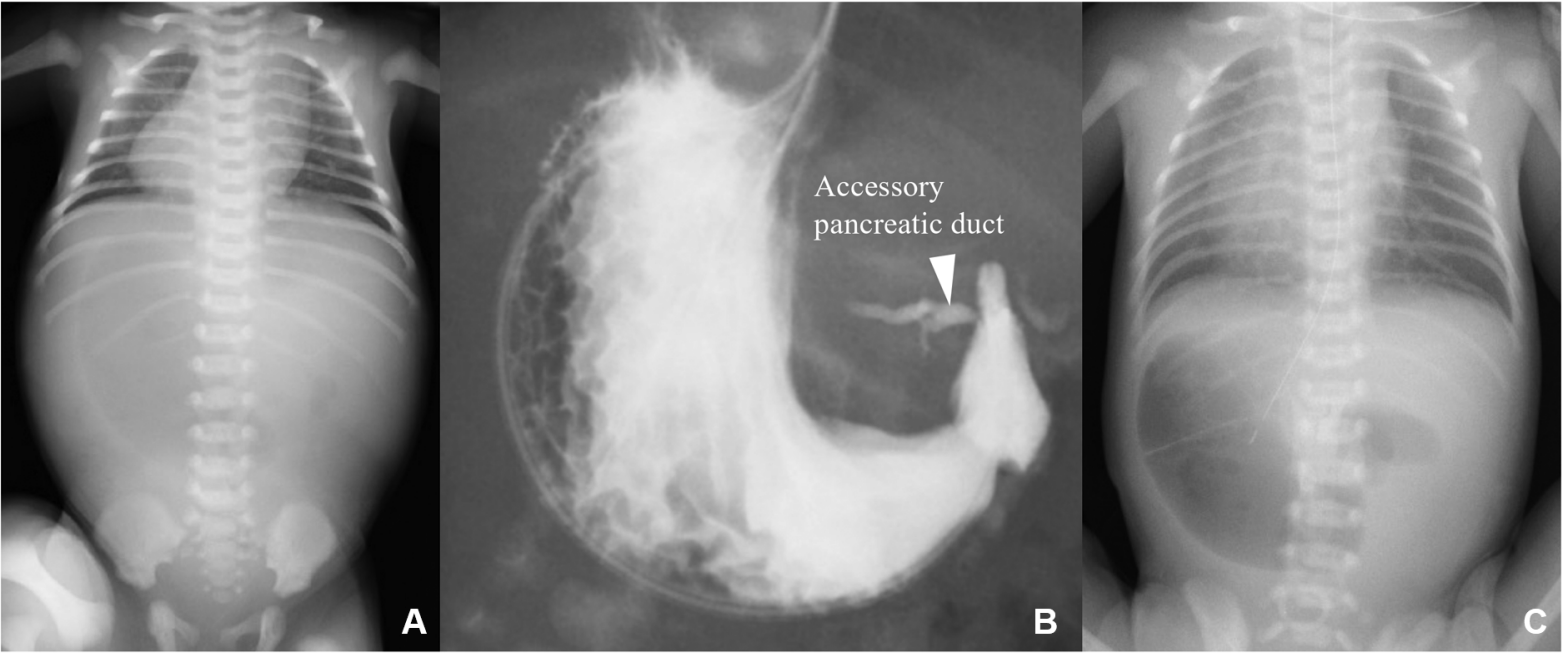
Preoperative imaging studies. (**A**) Chest radiograph showing the double-bubble sign with situs inversus. (**B**) Upper gastrointestinal contrast-enhanced imaging revealing duodenal atresia. The distal duodenum was enhanced through the accessory pancreatic duct. (**C**) Abdominal radiograph showing the double-bubble sign with situs inversus.

**Figure 2:**
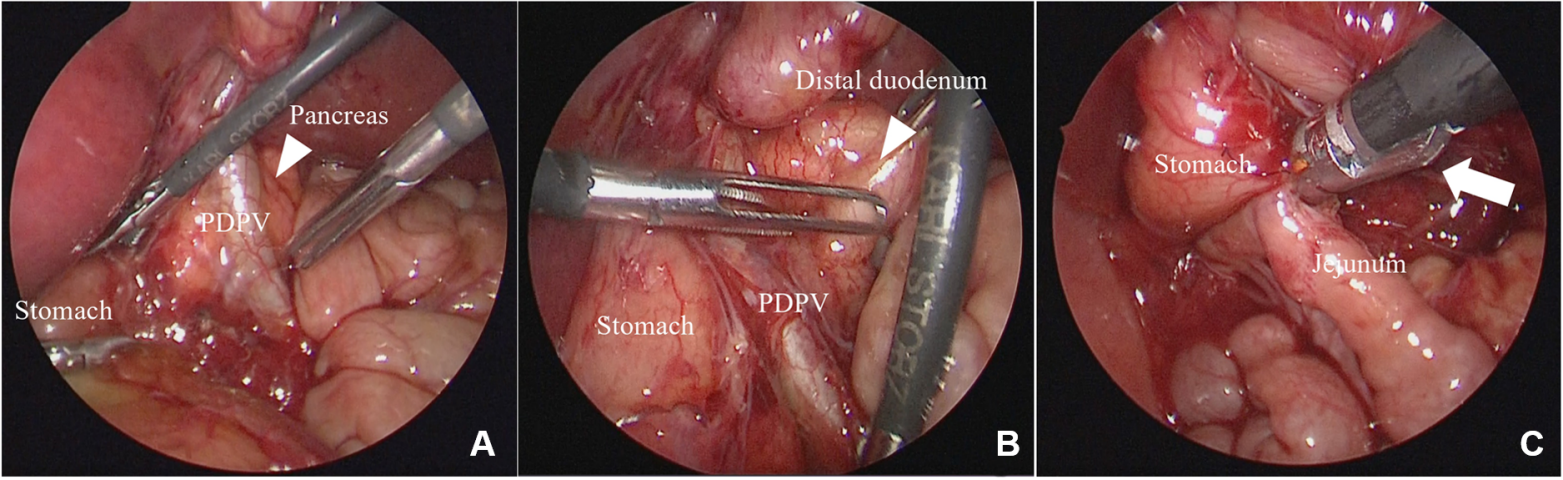
Laparoscopic findings for case 1. (**A**) The PDPV is confirmed. (**B**) The duodenum was obliterated near the pyloric end. (**C**) Gastrojejunostomy is performed using a 5 mm stapler (white arrow). PDPV, preduodenal portal vein.

Case 1: A 2-day-old boy with a gestational age of 38 weeks and weighing 3,200 g was transferred to our hospital with suspected duodenal atresia. The patient was prenatally diagnosed with situs inversus. Abdominal radiography revealed a double-bubble sign with situs inversus (Figure 1A). Upper gastrointestinal contrast imaging revealed duodenal atresia and a minor duodenal papilla in the oral duodenum (Figure 1B). Ultrasonography revealed situs inversus without any apparent cardiac anomalies or malrotation. The patient was diagnosed with duodenal atresia, and laparoscopic surgery was performed. Laparoscopy revealed situs inversus and duodenal atresia with a PDPV (Figures 2A,B). A laparoscopic gastrojejunostomy was performed using a 5 mm stapling instrument (Figure 2C). The postoperative course was uneventful, and the patient showed no apparent symptoms four years after surgery.

**Figure 3:**
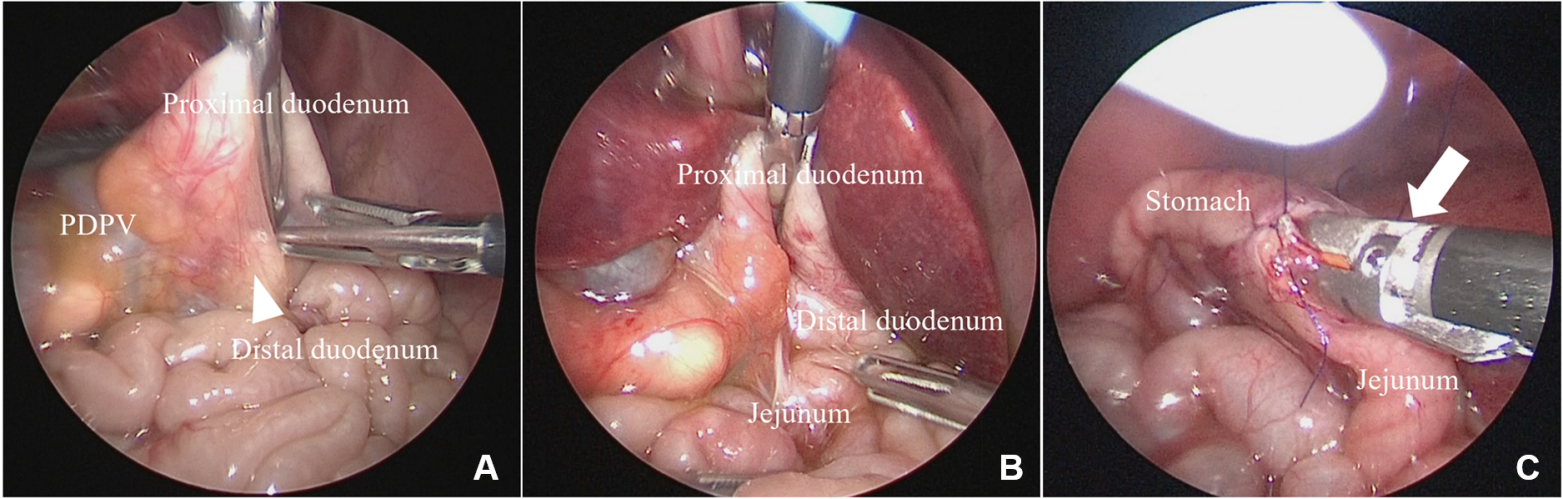
Laparoscopic findings for case 2. (**A,B**) A PDPV and duodenal atresia with an annular pancreas were diagnosed. (**C**) Gastrojejunostomy was performed using a 5 mm stapler (white arrow). PDPV, preduodenal portal vein.

Case 2: A 1-day-old girl with a gestational age of 37 weeks and weighing 2,278 g was born via cesarean section. The patient was prenatally diagnosed with situs inversus, polysplenia, and cardiac anomalies. Abdominal radiography revealed a double-bubble sign with situs inversus (Figure 1C). Duodenal atresia was suspected, and laparoscopic surgery was performed. Laparoscopy revealed situs inversus, annular pancreas, malrotation, and duodenal atresia with a PDPV (Figures 3A,B). A laparoscopic gastrojejunostomy was performed using a 5 mm stapling instrument (Figure 3C). The postoperative course was uneventful, and the patient remained under follow-up for two years after surgery with no apparent symptoms.

### 2.1. Surgical technique

#### 2.1.1. Port placement

**Figure 4:**
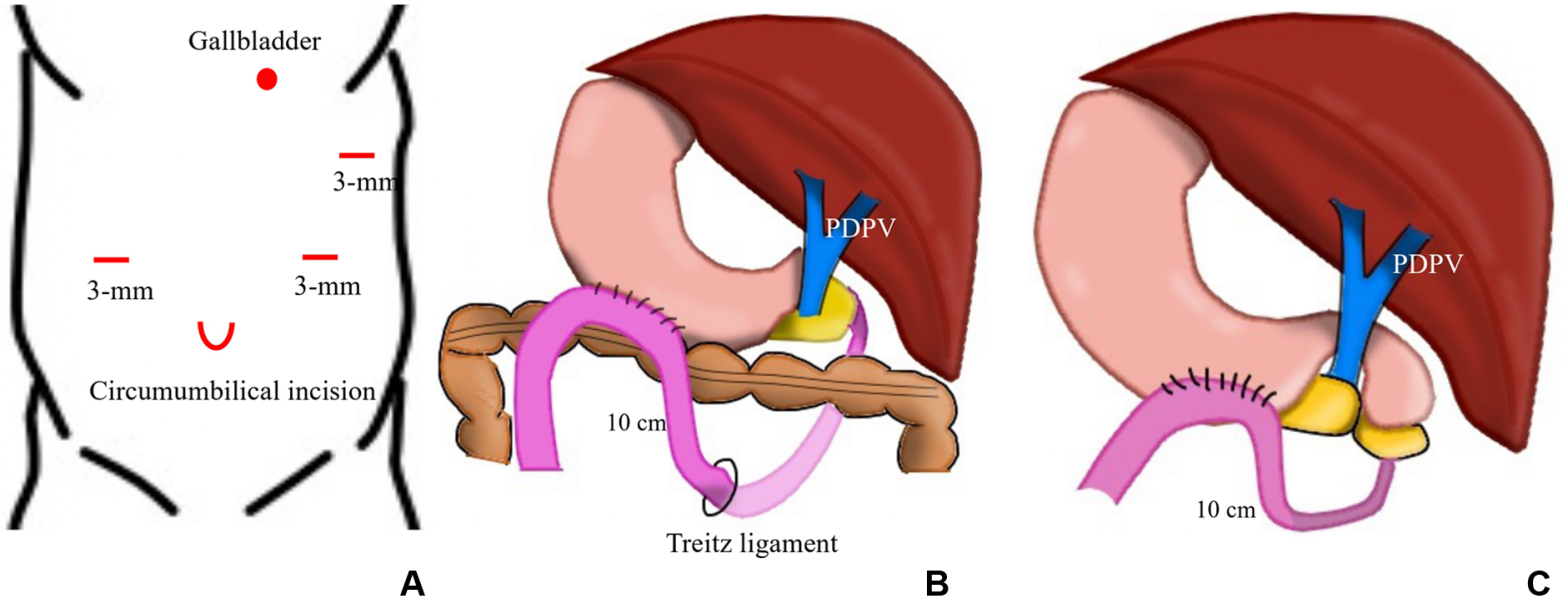
Schema of port placement and anastomosis. (**A**) A 5 mm balloon port for the laparoscope and three 3 mm trocars for the working port were placed at the umbilicus, right and left flanks, and left upper abdomen, respectively. (**B**) A laparoscopic gastrojejunostomy was performed about 10 cm from the ligament of Treitz in case 1. (**C**) Laparoscopic gastrojejunostomy was performed 10 cm from the duodenal atresia site in case 2.

A circumumbilical incision was made and a 5 mm balloon port was placed. The insufflation pressure was 8 mmHg with irrigation at 5 L/min. A 5 mm 30 degree laparoscope was inserted. Three 3 mm trocars were placed on the right flank, left flank, and left upper abdomen (Figure 4A).

#### 2.1.2. Laparoscopic manipulation

The gallbladder was pulled with 5-0 suture to the left upper abdomen to secure a field of view. The duodenal lumen was obliterated near the pyloric end and a PDPV was diagnosed. The anal portion of the duodenum was identified on the left side of the portal vein. Considering the anatomical position of the PDPV and the oral/anal side of the duodenum, duodenoduodenostomy might have obstructed the PDPV and kinked the pancreatic duct. Therefore, a laparoscopic gastrojejunostomy was performed. The posterior gastric wall and jejunum were anastomosed side to side with a 5 mm stapler, 10 cm from the ligament and 10 cm from the distal duodenum in cases 1 and 2, respectively. The anastomosis was performed via the antecolic route and antiperistalsis (Figures 4B,C).

## 3. Discussion

In this study, we selected laparoscopic gastrojejunostomy for cases of duodenal atresia with a PDPV and situs inversus, which resulted in good short- and long-term outcomes. A PDPV is a rare anomaly associated with situs inversus (5), which is often incidentally diagnosed during surgery.

A PDPV can cause extrinsic obstruction; however, less than 50% of PDPVs cause obvious obstruction (6). When a PDPV causes obstructive symptoms, a duodenoduodenostomy is performed to bypass the obstruction. In our two cases, the obstructive symptoms were apparently caused by duodenal atresia rather than the PDPV. Duodenoduodenostomy or jejunostomy is performed for duodenal atresia, even when accompanied by a PDPV. Gastrojejunostomy was selected by a process of elimination.

Gastrojejunostomies have several disadvantages. Although our literature search for reports on patients with duodenal atresia who underwent gastrojejunostomy failed to reveal detailed descriptions of long-term complications. Complications in adults with almost the same anatomical condition, that is, Billroth-II or Roux-en-Y reconstruction, have been reported. One such complication is afferent limb syndrome or blind loop syndrome, which occurs in approximately 1% of patients who undergo Billroth-II reconstruction (7) and 0.2% who undergo Roux-en-Y reconstruction (8) among patients who undergo distal gastrectomy. Braun anastomosis can prevent afferent limb syndrome; however, routine Braun anastomosis is controversial because of the relatively low incidence of afferent limb syndrome (7). We decided not to perform Braun anastomosis in our two cases because it would require an additional anastomosis. We will perform this procedure if the patients show symptoms of afferent limb syndrome in the future. Other complications include gastritis and bile reflux. Billroth-II reconstruction is more likely to cause gastritis and bile reflux than Roux-en-Y reconstruction (9). However, Roux-en-Y reconstruction requires two anastomotic sites, which results in a longer operation time and increase in anastomotic site complications. In addition, it causes Roux stasis syndrome in 7%–30% of cases (10–12). Considering the aforementioned factors, we believe that Billroth-II reconstruction was an appropriate reconstruction method for gastrojejunostomy in our cases. Another complication of gastrojejunostomy is marginal ulceration, which can be prevented with postoperative antacids (13).

Despite the disadvantages of gastrojejunostomy, we considered it a better option than duodenoduodenostomy or duodenojejunostomy in our cases, mainly because of the preservation of blood flow in the portal vein. Ohno et al. reported that the repeated passage of food over the anastomosis site, which overbridges the vessel, can induce thickening of the duodenal wall (14); therefore, they recommended loose overbridging duodenoduodenostomy to maintain portal blood flow. Loose overbridging duodenoduodenostomy was deemed difficult to perform in these two cases because the proximal duodenum was relatively short. We also considered that duodenoduodenostomy could cause kinking of the pancreatic duct as we have experienced four cases of pancreatitis in patients who had undergone duodenoduodenostomy for duodenal atresia/stenosis (15).

Importantly, gastrojejunostomy is not the best treatment option for other congenital duodenal obstructions. Moreover, long-term follow-up is essential for patients undergoing gastrojejunostomy, with routine check-ups for gastritis.

## 4. Conclusion

We encountered two cases of duodenal atresia with situs inversus and a PDPV. Although gastritis and bile reflux are risk factors, laparoscopic gastrojejunostomy was selected to preserve the portal vein blood flow. The appropriate surgical strategy is different for each patient and should be determined based on the patient's general and physical condition.

## Data Availability

The original contributions presented in the study are included in the article, further inquiries can be directed to the corresponding author.
